# Intraoperative Adrenal Insufficiency in a Patient with Prader-Willi Syndrome

**DOI:** 10.4021/jocmr1039w

**Published:** 2012-09-12

**Authors:** David W. Barbara, James D. Hannon, William R. Hartman

**Affiliations:** a Department of Anesthesiology, Mayo Clinic College of Medicine, Rochester, Minnesota, USA

**Keywords:** Prader-Willi syndrome, Adrenal insufficiency, Glucocorticoid, Corticosteroid, Steroid, Hypotension

## Abstract

Prader-Willi syndrome (PW) is a rare genetic disorder with multi-organ system involvement. These patients present many perioperative challenges including sleep-related breathing disorders, morbid obesity, thick salivary secretions, mental retardation, and difficult intravenous access. PW has been suggested to be associated with central adrenal insufficiency. We report a novel case of persistent severe hypotension from previously undiagnosed and asymptomatic adrenal insufficiency in a pediatric patient with Prader-Willi syndrome during spine surgery that resolved upon treatment with hydrocortisone.

## Introduction

Prader-Willi syndrome is a genetic condition characterized by neonatal hypotonia, mental retardation, developmental delay, kyphoscoliosis, hyperphagia resulting in obesity, short stature, hypogonadism, hypothalamic dysfunction, and characteristic facial appearance [1]. Its genetic basis is linked to a 15q11-13 deletion, which is inherited from the paternal chromosome in the majority of cases [1, 2]. The diagnosis is suspected based on clinical findings and confirmed with genetic testing. These patients frequently present to the operating room for spine and other orthopedic procedures [3, 4]. We describe a case of adrenal insufficiency in a patient with Prader-Willi syndrome undergoing spine surgery that resulted in persistent intraoperative hypotension requiring glucocorticoid replacement.

## Case Report

**Figure 1:**
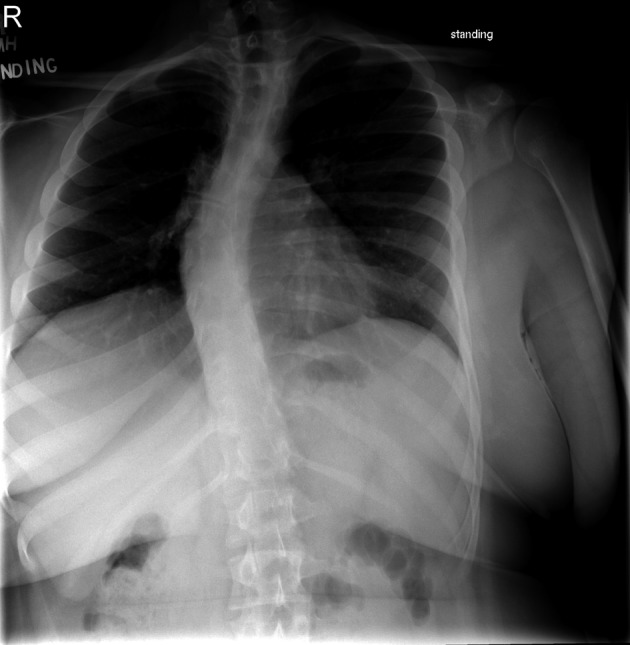
AP and lateral thoracolumbar spine X-Rays demonstrating S-shaped scoliosis of the cervical and thoracic spine with left lower cervical and upper thoracic curves and right mid and lower thoracic curves. Increased thoracic kyphosis and lumbar lordosis is present. There is mild anterior wedging of a mid thoracic vertebra.

A 16 year-old male with Prader-Willi syndrome weighing 109 kg (body mass index 41 kg/m^2^) presented for T1-L2 posterior instrumented spinal fusion due to progressive severe kyphoscoliosis (Fig. 1). His past medical history was significant for hypogonadism and sleep apnea that resolved after undergoing tonsillectomy and adenoidectomy. The patient was not taking any medications prior to surgery. Neurologic examination revealed normal strength and sensation in the upper and lower extremities. Preoperative pulmonary function testing revealed mild obstruction with forced vital capacity 4.0 L (88% predicted), forced expiratory volume in one second 3.0 L (77% predicted), and forced expiratory volume in one second to forced vital capacity ratio 75%. Prior to surgery, a pediatric endocrinology consult was obtained, and 100 mg of hydrocortisone was recommended upon induction of anesthesia, given the potential association of Prader-Willi with adrenal insufficiency. The patient previously had not been diagnosed with or had symptoms related to adrenal insufficiency. Preoperative hemoglobin was 12.6 g/dL.

General anesthesia was induced with intravenous fentanyl (250 μg), lidocaine (100 mg), propofol (100 mg), and succinylcholine (100 mg), and the trachea was easily intubated with a 7 mm endotracheal tube. Two large bore peripheral IVs (18 g and 16 g), a left radial arterial line, and a right internal jugular triple lumen 7 French central line were placed. Anesthesia was maintained with isoflurane, fentanyl, and hydromorphone. Muscle relaxant was not utilized as motor evoked potentials, somatosensory evoked potentials, and electromyography was monitored. On induction, hydrocortisone 100 mg was administered intravenously, and the patient was positioned prone for the procedure.

Approximately 7 hours into the surgical procedure, the patient developed persistent hypotension (persistent systolic pressures around 80 mmHg and diastolic pressures around 50 mmHg). Phenylephrine (800 μg), calcium chloride (300 mg), 4 L crystalloid, 500 mL 5% albumin, 1 unit packed red blood cells, 300 mL cell saver, and 300 mL fresh frozen plasma were administered without resolution of the hypotension. Intraoperative nadir hemoglobin was 9.8 g/dL. With persistent refractory hypotension despite attempted volume resuscitation and multiple vasopressors, additional glucocorticoid supplementation was administered in the form 50 mg of intravenous hydrocortisone at hour 9 of surgery. Resolution of the hypotension was promptly noted at approximately the 10th hr of surgery. Estimated blood loss for the procedure was approximately 3000 mL, and urine output was 4569 mL.

Postoperatively, the patient remained intubated and was transferred to the intensive care unit for further monitoring. On day 8 he was successfully discharged home from the hospital.

## Discussion

Initially described in 1956, Prader-Willi syndrome has an estimated incidence of 1:10,000 - 1:52,000 and a reported male predominance [4-8]. Neonatal hypotonia, poor feeding requiring special assistance, and growth restriction are hallmarks of Prader-Willi syndrome in the affected neonate and infant [1, 3, 9]. Characteristic facial appearance consists of infantile dolichocephaly (disproportionately longer and narrower head size), narrow face, small mouth with down-turned corners, thin superior lip, and almond-shaped eyes [1]. Hypogonadism, developmental delay, and mental retardation may be present. After 12 months of age, the infantile feeding difficulties change to hyperphagia, weight gain, and obsession with food, resulting in obesity if not regulated. Additional features may include non-insulin dependent diabetes mellitus, extremely viscous saliva, sleep-related breathing disorders, and infantile temperature instability [1, 6, 10]. The death rate has been estimated at 3% per year, with commonly reported mortality causes consisting of respiratory failure, cor pulmonale, and infantile aspiration [7, 8, 11]. Consensus diagnostic scoring criteria exist to guide the clinician in obtaining appropriate genetic confirmation of the diagnosis or diagnosing the syndrome if genetic testing is not available [1, 9].

One case series on pediatric patients with this syndrome reported 71% of patients having spinal deformity [4]. In addition to surgery of the spine, patients with Prader-Willi may present for orchidopexy, dental procedures, and tonsillectomy, making a thorough understanding of the multiorgan involvement essential to successful management [3, 12].

Prader-Willi syndrome presents unique challenges to physicians in the perioperative period. Infantile hypotonia may result in spontaneous respirations being inadequate, necessitating mechanical ventilation [3]. Extrinsic restrictive pulmonary disease from spinal abnormalities can lead difficulty with mechanical ventilation, and this coupled with sleep-related breathing disorders make the patient more likely to have apnea and hypoventilation postoperatively and with preoperative sedation [3, 4, 12]. Both central and obstructive sleep apnea has been noted to occur with Prader-Willi syndrome even in the absence of obesity [1, 3, 12, 13]. Antisialagogues such as atropine, glycopyrrolate, and scopolamine are not advised given the thick oral secretions in these patients [3]. Morbid obesity may complicate intravenous access and anesthetic management; however, airway difficulty has not been noted [3, 14]. Previous case reports and small case series have described the anesthetic management of these patients, but none have reported intraoperative hypotension refractory to volume resuscitation and vasopressors that was successfully treated with glucocorticoids [3, 12].

Central adrenal insufficiency is noted to affect as many as 60% of Prader-Willi patients when stressed [15]. Interestingly, these patients have normal cortisol levels in the absence of stress. Several authors have hypothesized that unrecognized central adrenal insufficiency may explain the high annual death rate in patients with this syndrome [14, 15]. Even in patients without Prader-Willi syndrome who may have iatrogenic adrenal insufficiency from exogenous glucocorticoids, controversy exists whether perioperative steroids are necessary [16, 17]. Nonetheless, case reports exist of patients who, despite receiving the same dose of stress dose steroids upon induction that our patient received, required additional intraoperative glucocorticoids for acute Addisonian crisis manifested by severe refractory hypotension [18]. We hypothesize that with the continual stress of major spine surgery, our patient developed adrenal insufficiency that was undertreated with the initial dose of hydrocortisone given seven hours prior to the onset of severe hypotension. The refractoriness of his hypotension to repletion of intravascular volume deficits, additional increases in preload, and multiple vasopressors combined with the resolution of the hypotension with additional hydrocortisone doses make adrenal insufficiency the likely etiology of hypotension.

In conclusion, Prader-Willi patients present many perioperative challenges to anesthesiologists and surgeons. This case illustrates the importance of understanding the multiorgan clinical features of the syndrome and recognizing the possibility of adrenal insufficiency contributing to severe hypotension during periods of stress such as with surgery. For major surgery, glucocorticoids should be considered as prophylaxis or, at very least, be readily available should refractory hypotension or other signs of adrenal insufficiency develop.
